# The Sessional Medical Addresses

**Published:** 1912-10-05

**Authors:** 


### THE SESSIONAL MEDICAL ADDRESSES.

At a time when the doubts and difficulties of the voluntary hospitals and of their medical schools created by the National Insurance Act grow thicker and darker like gathering clouds on the horizon of the future, it is not surprising that a tone of unusual gravity pervades the addresses delivered at the opening of the sessions on October 1. Medical education has long been drifting in England into a state which no one can regard as satisfactory. In these circumstances it is only natural that the Insurance Act and its far-spreading ramifications should bulk largely in the sessional addresses.

The Lord Mayor of London, Sir Thomas Crosby, M.D., not only addressed the students at the formal opening of the session at the St. Mary's Hospital School, but also later on presided at the dinner of the St. Thomas's Hospital Old Students' Association. He expressed the belief that the medical profession is not receiving fair treatment at the hands of the Government; and at the close of a year of office such as his has been this expression of Sir Thomas Crosby's mind must surely carry the greatest possible weight in circles which are not much in sympathy with the opposition to the Act in other respects. At St. Bartholomew's Sir Wm. Osler touched upon the same theme with his accustomed virility of thought and happiness of expression. He emphasised especially the new-found solidarity, evoked by the Act, of the medical profession, and expressed the conviction that never again would the doctors submit to be sweated as they have been in the past. He also touched upon the problem of the acceptance of payments from patients for hospital treatment. Doubtless by reason of his Canadian and American upbringing, Sir Wm. Osler is in favour of accepting this principle, which the ultimate workings of the Insurance Act are bound to bring into prominence in a few years.

At St. George's Hospital Mr. H. B. Grimsdale made a furious onslaught upon the Act, or rather upon the Government for its methods in connection with the Act. Mr. Grimsdale's biting sarcasms revealed him as a bitter opponent of the Chancellor over the terms and treatment which the medical profession is expected to swallow; but his address revealed argument and careful study as well as invective, and formed an excellent summing-up of the case for the profession in the present struggle. In the evening Mr. E. B. Turner also dealt with the Act, and stated that the draft regulations (now supposed to be near completion and publication) were as a whole quite unsatisfactory and unworkable from the British Medical Association's point of view at the time when Mr. Turner resigned his position on the Advisory Committee. Mr. Turner's position as one of the foremost champions in London of the medical practitioner against the Chancellor, and his pointed allusions to the regulations in the presence of Sir Clifford Allbutt and Dr. Latham (both of whom refused to resign from the Committee when requested to do so by the British Medical Association), rendered these references to the regulations distinctly *piquant*.

Next comes Miss Jane Walker's address to the women students at the Royal Free Hospital. Dr. Walker touched upon many topics, from hospital letters to race suicide. Upon the latter subject the published report of the address does not convey very clearly what her sentiments are; for in one passage she denounced—very rightly, as all must admit—the numerous advertisements which appear for married couples "without encumbrance," and in another she is reported to have entered a plea for quality as opposed to quantity in the next generation.

Finally, we may note Dr. Walker's picturesque proposal of a bonfire to celebrate the King's birthday, composed of hospital letters, to be organised by the King's Fund. From which it will be seen that at the Royal Free Hospital, no less than at the men's schools in London, topics of the day are handled freely and fearlessly by those who deliver the annual sessional addresses.

